# Supplementary material for: ORAI2 modulates store-operated calcium entry and T cell-mediated immunity
Source: Nat Commun. 2017 Mar 15;8:14714. doi: 10.1038/ncomms14714 (PMC5355949; doi:10.1038/ncomms14714)
Supplement: Supplementary Information — Supplementary Figures 1-9 and Supplementary Tables 1-3 [file ncomms14714-s1.pdf]

Supplementary Figure 1

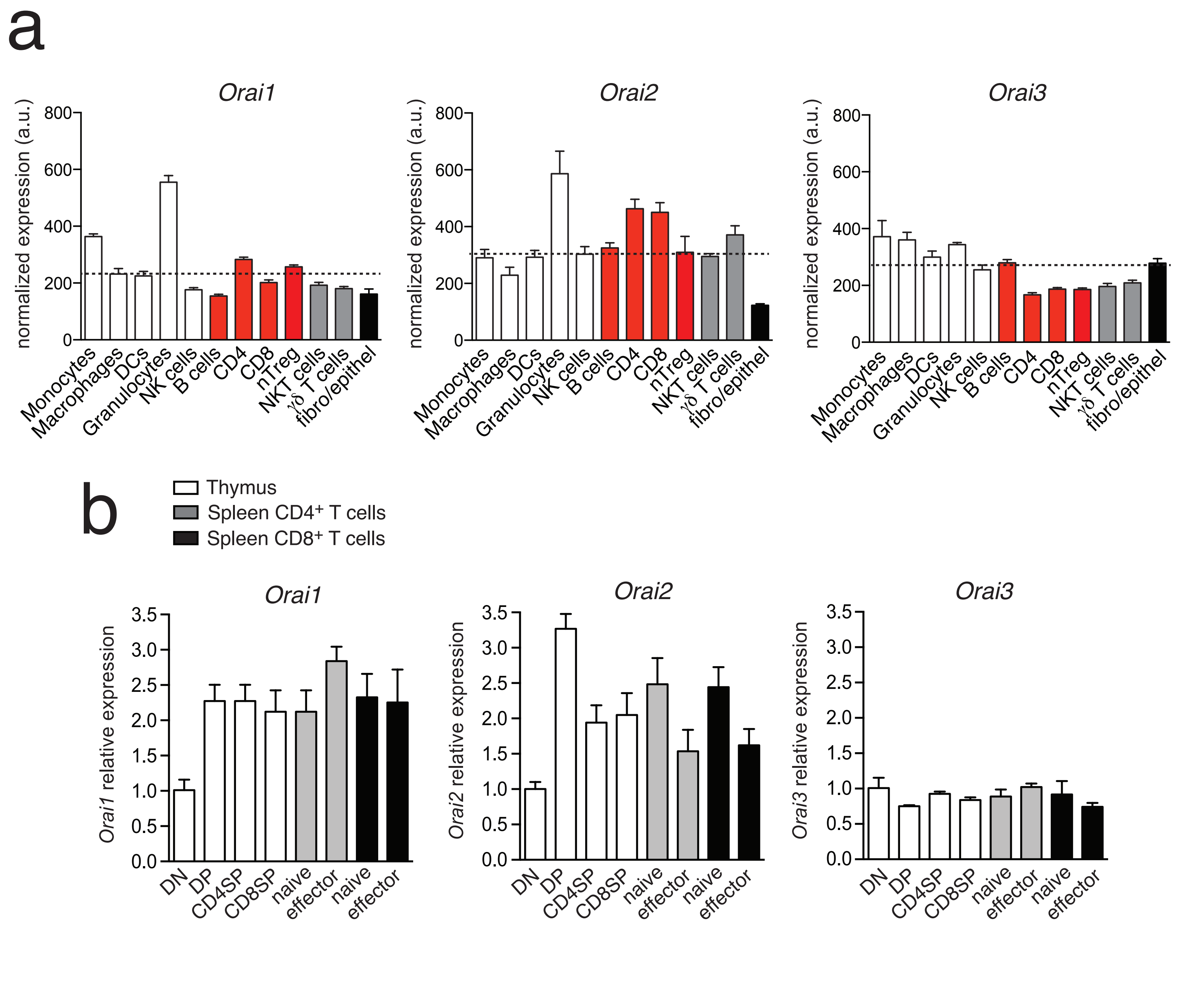

Supplementary Figure 1. Expression of ORAI1, ORAI2 and ORAI3 in immune cells. (a) Expression of *Orai1, Orai2 and Orai3* genes in different immune cell subsets. Analysis of microarray data from the ImmGen database (*ImmGen.org*). **(b)** Analysis of *Orai1, Orai2* and *Orai3* gene expression in FACS-sorted $CD4^{-}CD8^{-}$ (DN, double negative), $CD4^{+}CD8^{+}$ (DP, double positive), $CD4^{+}CD8^{-}$ (CD4SP $CD4^{+}$ single positive) and $CD4^{-}CD8^{+}$ (SD8SP, $CD8^{+}$ single positive) thymocytes and $CD62L^{hi}CD44^{lo}$ (naïve) and $CD62L^{lo}CD44^{hi}$ (effector) peripheral $CD4^{+}$ or $CD8^{+}$ T cells using qRT-PCR; means ± SEM of 4 mice.

Supplementary Figure 2

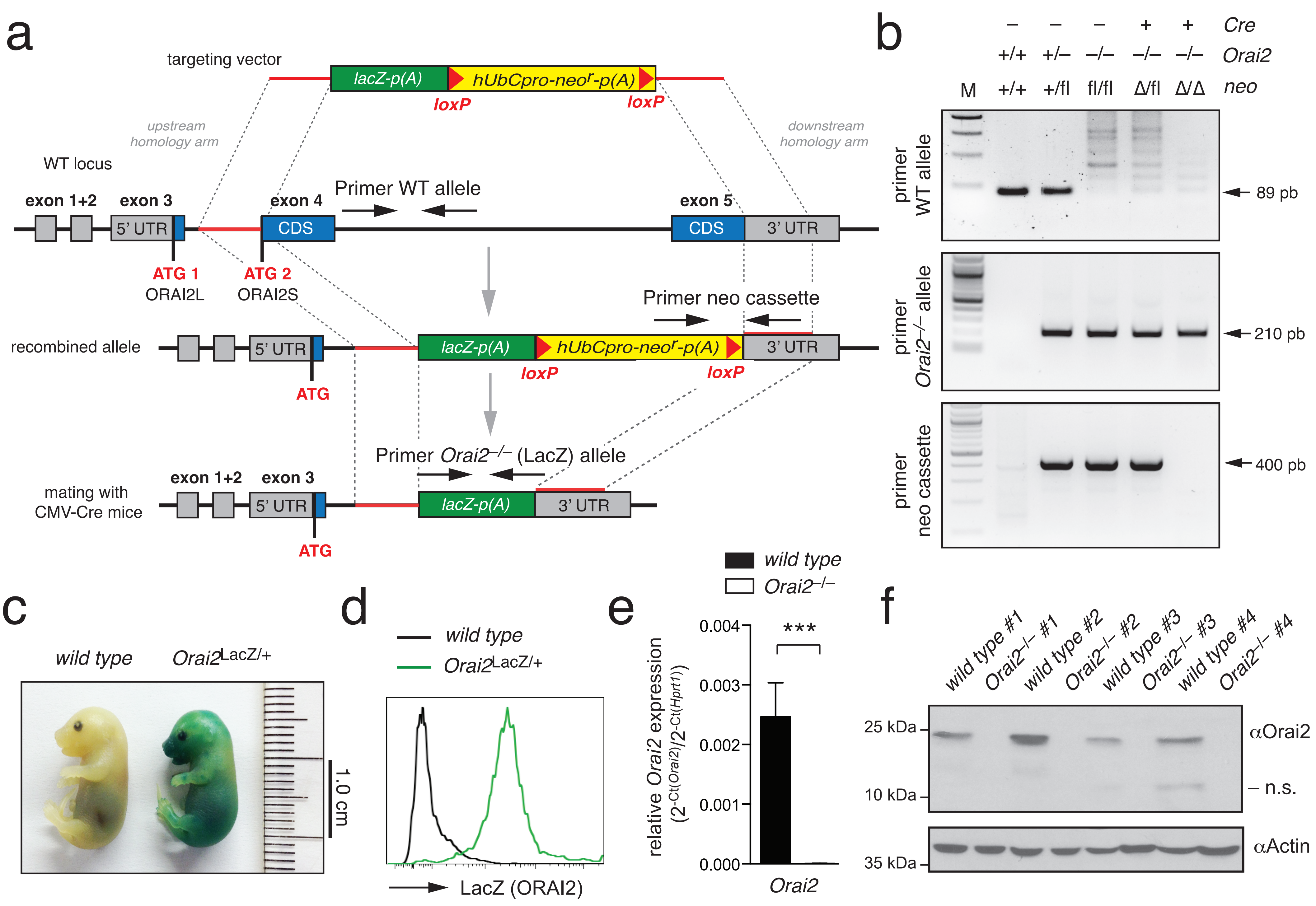

Supplementary Figure 2. Generation of *Orai2*-deficient mice. (a) Targeting strategy to disrupt *Orai2* gene expression. Exon 4 and the 5' region of exon 5 encoding the coding sequence of the *Orai2* gene were replaced by homologous recombination with a loxP-flanked neomycin selection cassette (neo^R) and a LacZ reporter construct. The neo^R cassette was later removed by mating $Orai2^{LacZ/+}$ offsprings to CMV-Cre mice, followed by removal of the Cre transgene by backcrossing $Orai2^{LacZ/+}$ mice to C57BL/6 mice. Primers for genotyping of $Orai2^{-/-}$ mice are indicated; see also materials and methods. **(b)** Representative genotyping results using primers specific for the WT allele, the recombined *Orai2* allele and the loxP-flanked neomycin resistance cassette before (fl/fl) and after deletion (Δ/Δ) by CMV-cre. **(c)** Analysis of global LacZ expression in $Orai2^{LacZ/+}$ tissue by FDG staining of whole E17.5 embryos; scale bar represents 1 cm. **(d)** Detection of LacZ activity in $Orai2^{LacZ/+}$ bone marrow derived macrophages (BMDMs) by flow cytometry using the fluorescent LacZ substrate FDG. **(e)** Analysis of *Orai2* expression in WT and $Orai2^{-/-}$ BMDMs by qRT-PCR; means ± SEM of 3 mice. **(f)** ORAI2 protein expression in WT and $Orai2^{-/-}$ BMDMs by immunoblot analysis; BMDMs from 4 WT and 4 $Orai2^{-/-}$ mice were analyzed. ***, $p<0.001$ in (e) using unpaired Student's t test.

Supplementary Figure 3

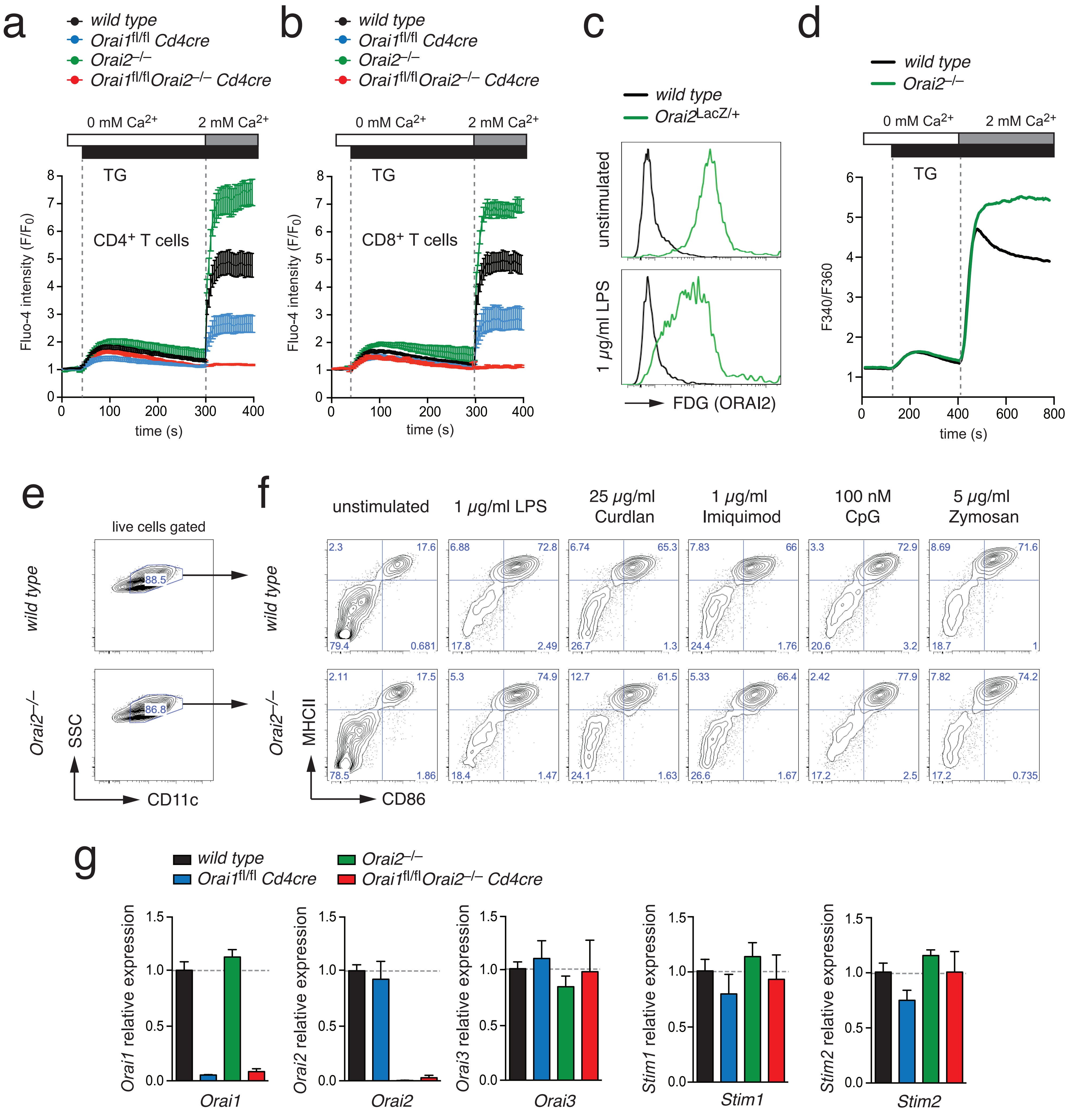

Supplementary Figure 3. Increased SOCE in naïve T cells and BMDCs from *Orai2*$^{-/-}$ mice is not due to compensatory upregulation of other CRAC channel genes. (a,b) Analysis of SOCE in CD4^{+} (a) and CD8^{+} T cells (b) isolated from WT, *Orai1*$^{fl/fl}$*Cd4cre*, *Orai2*$^{-/-}$ and *Orai1*$^{fl/fl}$*Orai2*$^{-/-}$*Cd4cre* (DKO) mice. T cells were loaded with Fluo-4 and analyzed by flow cytometry. 1 μM thapsigagrgin (TG) and 2 mM extracellular Ca^{2+} were added as indicated. Means ± SEM of 4-7 mice. **(c)** LacZ expression (*Orai2* reporter) in unstimulated and LPS-stimulated *Orai2*$^{LacZ/+}$ bone marrow-derived dendritic cells (BMDCs). **(d)** Increased SOCE following thapsigargin (TG) stimulation in BMDCs from WT and *Orai2*-deficient (*Orai2*$^{-/-}$) mice using a FlexStation plate reader. **(e,f)** Normal differentiation of WT and *Orai2*-deficient (*Orai2*$^{-/-}$) CD11c^{+} BMDCs after 8 days of GM-CSF culture **(e)** and normal upregulation of MHCII and CD86 **(f)** after 24 h stimulation with various microbial stimuli. **(g)** Analysis of *Stim1, Stim2, Orai1, Orai2* and *Orai3* gene expression in WT, *Orai1*$^{fl/fl}$*Cd4cre*, *Orai2*$^{-/-}$ and DKO CD4^{+} T cells using qRT-PCR; means ± SEM of 3 mice.

Supplementary Figure 4

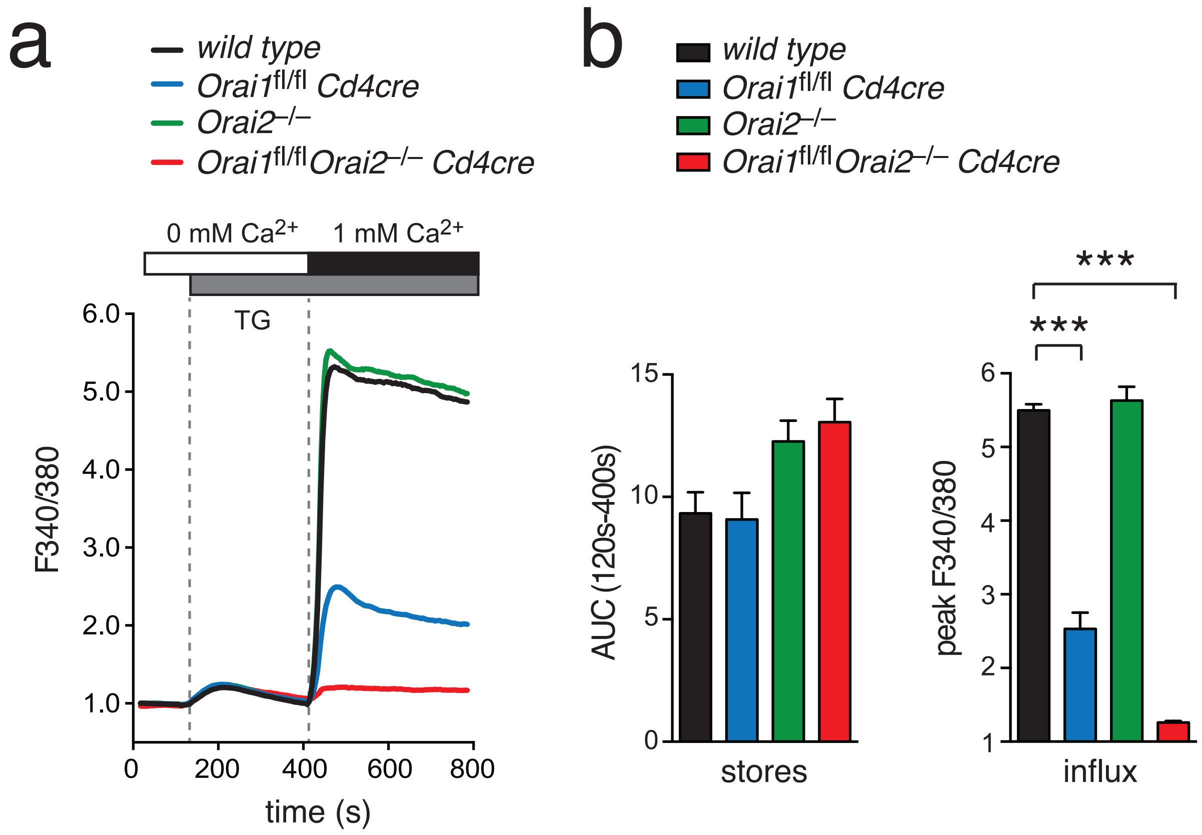

Supplementary Figure 4. Normal SOCE in *in vitro* differentiated *Orai2*$^{-/-}$ effector CD4$^+$ T cells. (a) SOCE in CD4$^+$ T cells isolated from WT, *Orai1*$^{fl/fl}$*Cd4cre*, *Orai2*$^{-/-}$ and *Orai1*$^{fl/fl}$*Orai2*$^{-/-}$*Cd4cre* (DKO) mice, stimulated with anti-CD3/CD28 and cultured for 3 days *in vitro* in the presence of 50 U/ml rhIL-2. Analysis of Ca^{2+} store depletion and SOCE following thapsigargin (TG) stimulation and SOCE after re-addition of 1 mM extracellular Ca^{2+} in differentiated T cells using a FlexStation plate reader. **(b)** Quantification of Ca^{2+} released from ER stores in the absence of extracellular Ca^{2+} (area under the curve, $AUC_{120s\text{-}400s}$ of F340/380) and SOCE (peak F340/380) as shown in (a). Means ± SEM of 4 mice. ***, $p<0.001$ in (b) using unpaired Student's t tests.

Supplementary Figure 5

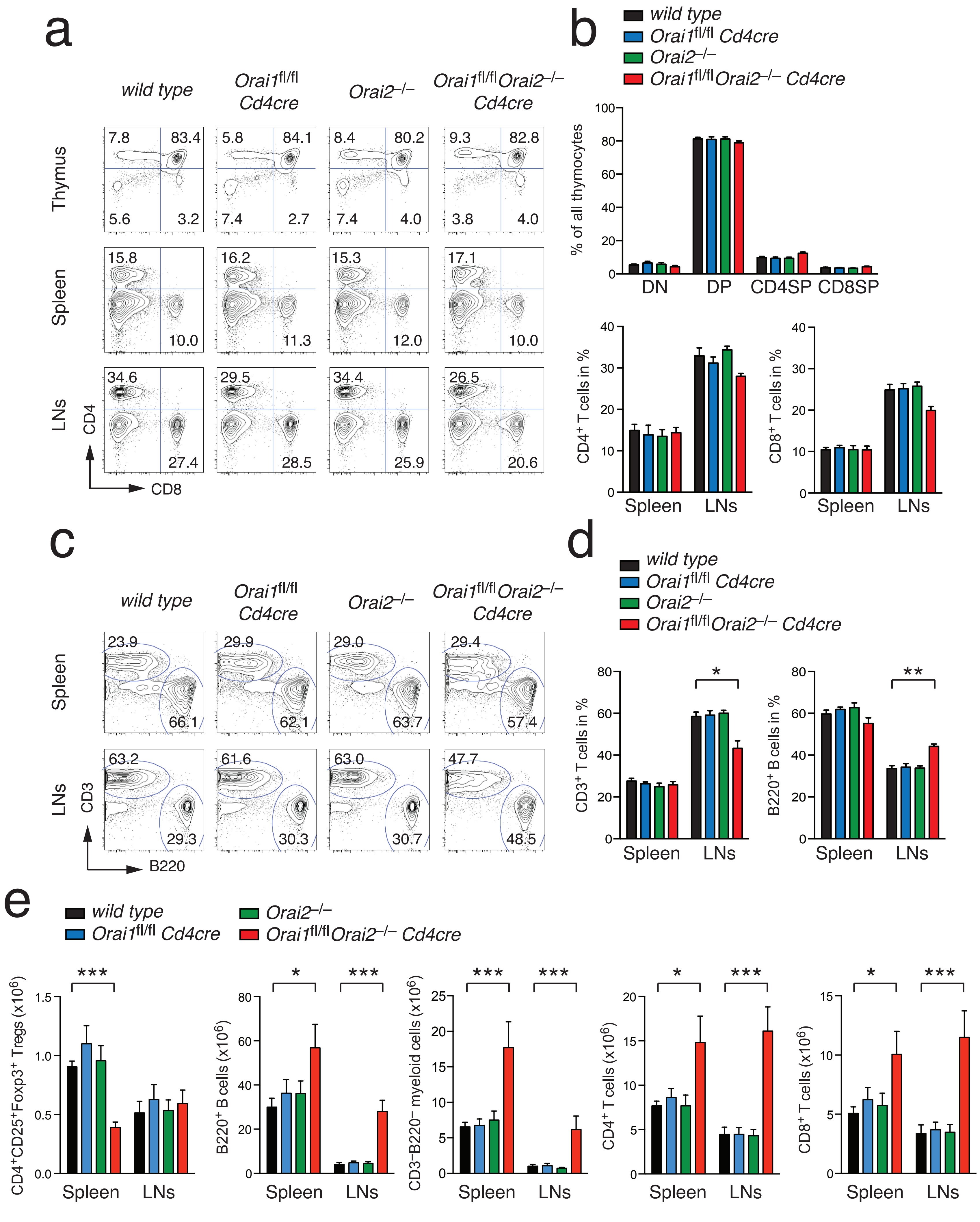

Supplementary Figure 5. Lymphocyte populations in *Orai1$^{fl/fl}$Orai2$^{-/-}$Cd4cre* mice. (a) Analysis of CD4$^-$CD8$^-$ (DN, double negative), CD4$^+$CD8$^+$ (DP, double positive), CD4$^+$CD8$^-$ (CD4SP, CD4$^+$ single positive) and CD4$^-$CD8$^+$ (CD8SP, CD8$^+$ single positive) thymocytes and CD4$^+$ and CD8$^+$ T cells subsets in spleen and LNs of WT, *Orai1$^{fl/fl}$Cd4cre*, *Orai2$^{-/-}$* and *Orai1$^{fl/fl}$Orai2$^{-/-}$Cd4cre* (DKO) mice by flow cytometry. **(b)** Quantification of thymic and peripheral T cell populations as shown in (a); means ± SEM of 7-11 mice. **(c)** Analysis of CD3$^+$ T cell and B220$^+$ B cell populations in spleen and LNs of WT, *Orai1$^{fl/fl}$Cd4cre*, *Orai2$^{-/-}$* and DKO mice by flow cytometry. **(d)** Quantification of T and B cell populations as shown in (c); means ± SEM of 9-11 mice. **(e)** Absolute cell numbers of CD4$^+$CD25$^+$Foxp3$^+$ Tregs, B220$^+$ B cells, CD4$^+$ and CD8$^+$ T cells and B220$^-$CD3$^-$ myeloid cells as shown in Fig. 5c and (a-d) in spleen and LNs; means ± SEM of 7-11 mice. *, p<0.05; **, p<0,005; ***, p<0.001 in (d,e) using unpaired Student's t tests.

Supplementary Figure 6

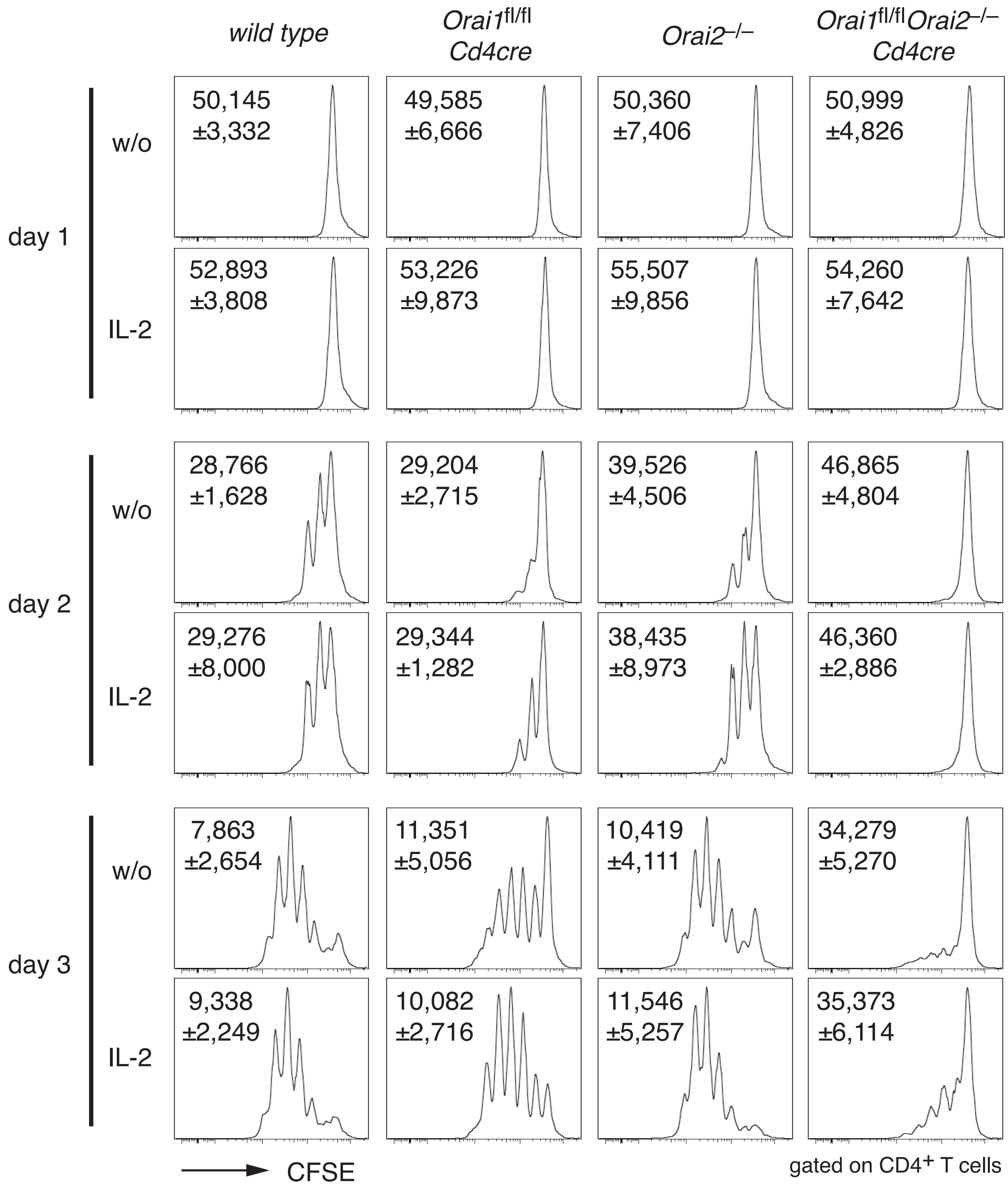

Supplementary Figure 6. *Orai1/Orai2*-deficient T cells show defective proliferation *in vitro*. $CD4^+$ T cells were isolated from WT, *Orai1*$^{fl/fl}$*Cd4cre*, *Orai2*$^{–/–}$ and *Orai1*$^{fl/fl}$*Orai2*$^{–/–}$*Cd4cre* (DKO) mice, loaded with CFSE, stimulated with plate-bound anti-CD3/CD28 and cultured for 1-3 days *in vitro* with or without 50 U/ml rhIL-2. Proliferation was analyzed by CFSE dilution using flow cytometry; mean fluorescence intensity (MFI) ± SEM of CFSE from 2-3 experiments is shown in the histograms. Proliferation data from day 4 are shown in Fig. 6a.

Supplementary Figure 7

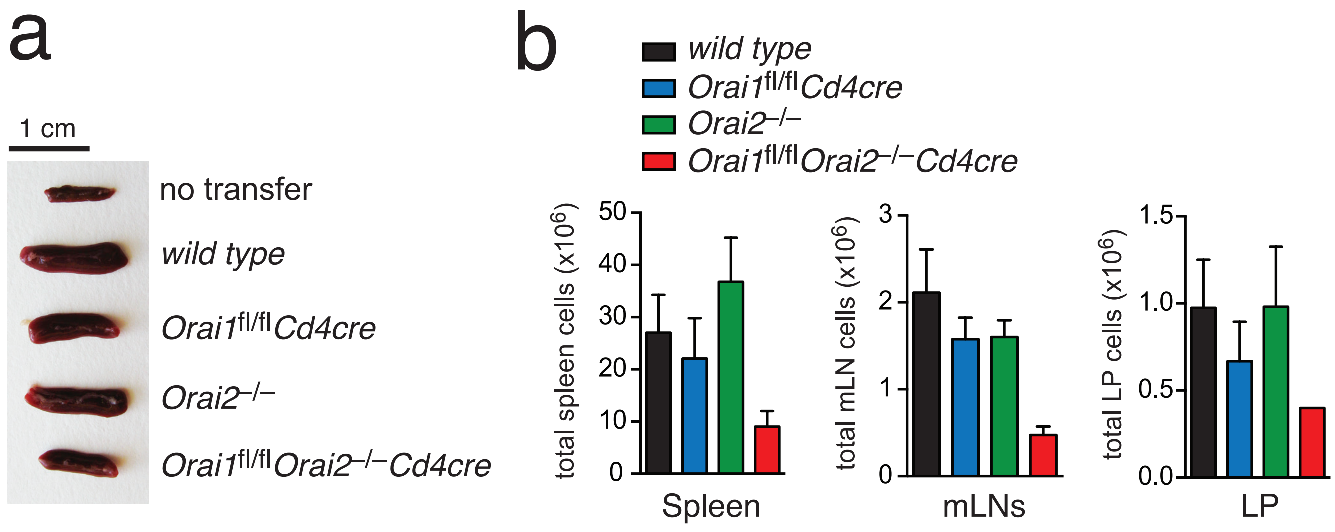

Supplementary Figure 7. Impaired proliferation of *Orai1/Orai*2-deficient T cells in lymphopenic host mice. (a) Representative pictures of spleens of *Rag1*$^{-/-}$ host mice 12 weeks after adoptive transfer of 5x10^5 CD4$^+$CD25$^-$CD62L^{hi} naïve T cells from WT, *Orai1*$^{fl/fl}$*Cd4cre*, *Orai2*$^{-/-}$ and *Orai1*$^{fl/fl}$*Orai2*$^{-/-}$*Cd4cre* (DKO) mice; scale bar represents 1 cm. **(b)** Absolute cell numbers in the spleens, mesenteric lymph nodes (mLNs) and lamina propria (LP) of *Rag1*$^{-/-}$ host mice 12 weeks after transfer of 5x10^5 CD4$^+$CD25$^-$CD62L^{hi} naïve T cells.

Supplementary Figure 8

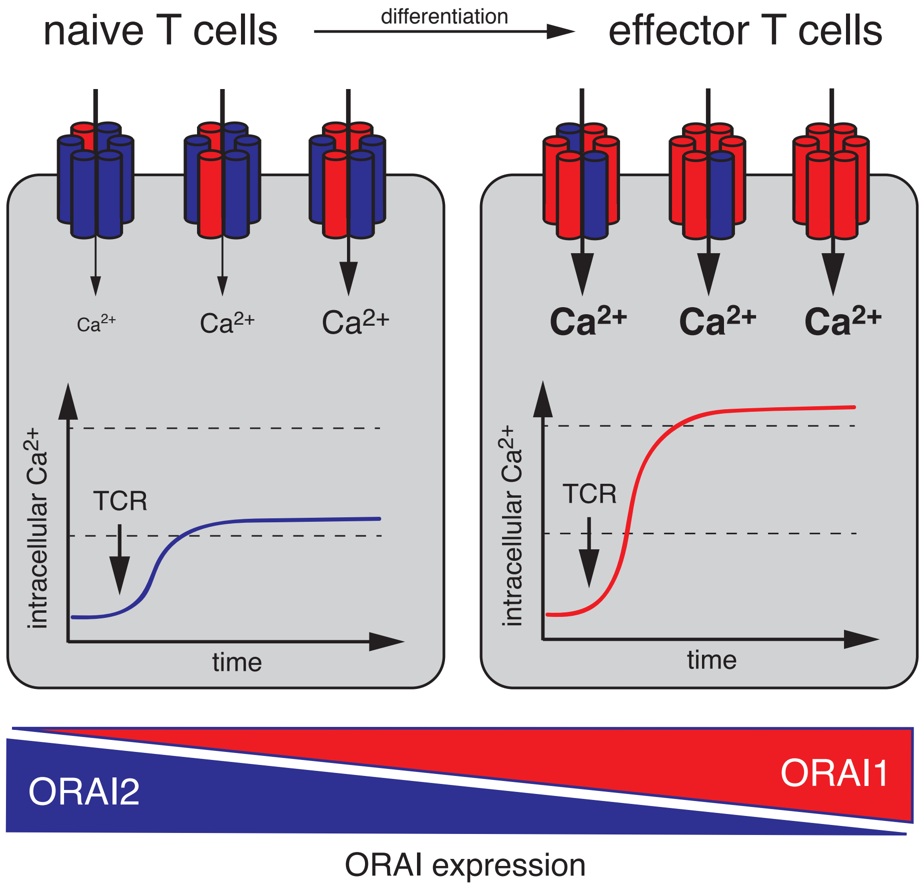

Supplementary Figure 8. Model of SOCE modulation in T cells by the stoichiometry of ORAI1 and ORAI2 in the CRAC channel complex. Naive T cells co-express ORAI1 and ORAI2, which together form the CRAC channel. Antigen experienced effector T cells in the spleen of lymph nodes or T cells stimulated and cultured *in vitro* upregulate expression of ORAI1 and downregulate ORAI2, resulting in an increased ORAI1:ORAI2 ratio and CRAC channels predominantly formed by ORAI1 subunits. The change in ORAI1:ORAI2 stoichiometry in effector T cells results in increased SOCE compared to naive T cells. ORAI2 attenuates the function of ORAI1 and SOCE when both subunits are co-expressed and form an ORAI1:ORAI2 heteromeric channel. The reciprocal expression of ORAI1 and ORAI2 in naïve versus effector T cells provides a mechanism to fine-tune the magnitude of SOCE and thus the strength of T cell-mediated immune response.

Supplementary Figure 9

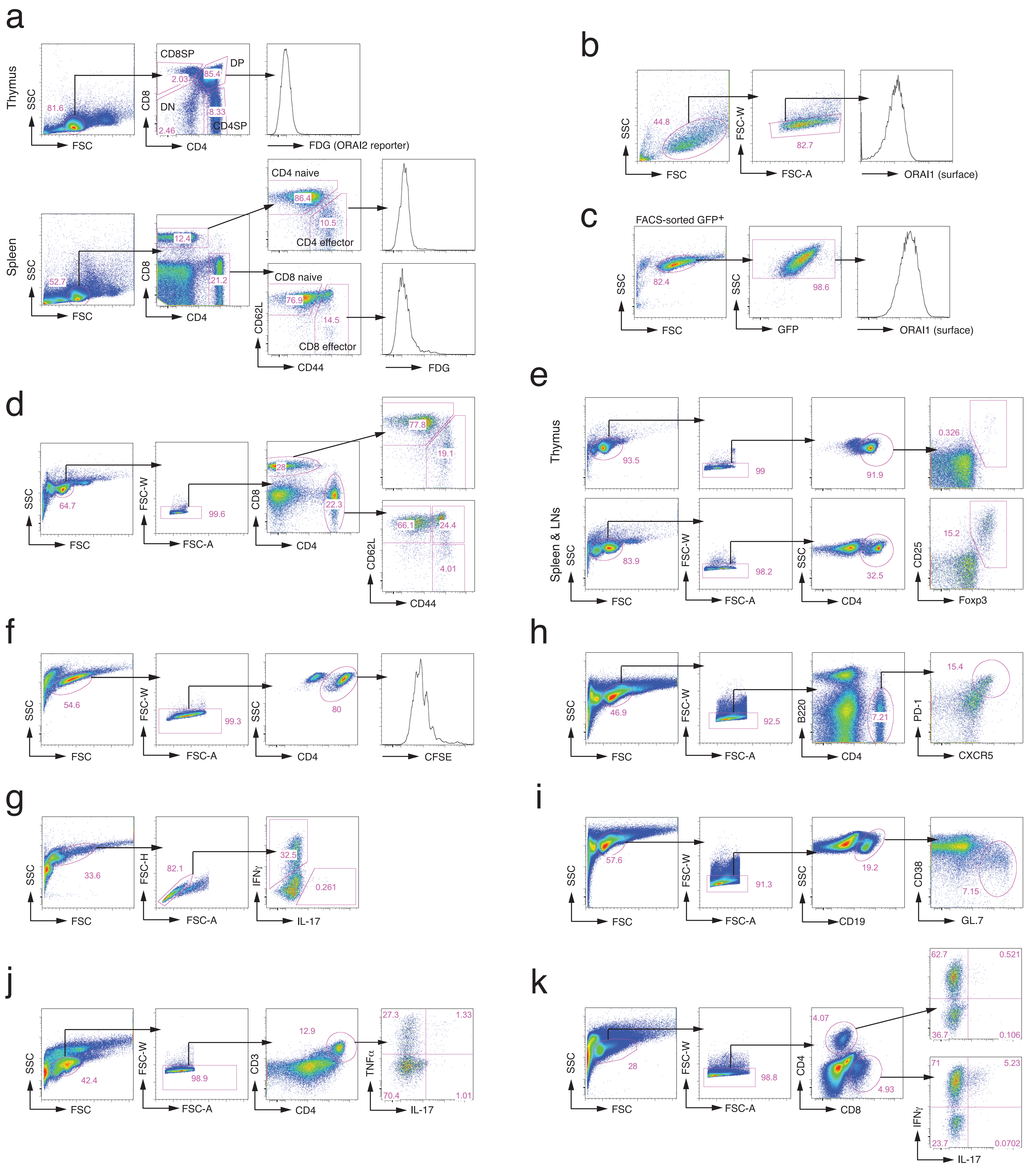

Supplementary Figure 9. Gating strategies for flow cytometry analyses. Representative gating strategies to analyze: **(a)** ORAI2 expression using the LacZ substrate FDG as shown in Fig. 1a,b; **(b)** ORAI1 surface expression as shown in Fig. 2g; **(c)** ORAI1 surface expression as shown in Fig. 4c; **(d)** Naïve and effector $CD4^+$ and $CD8^+$ T cell subsets in spleen and LNs as shown in Fig. 5b; **(e)** Treg cells in the thymus and peripheral lymphoid organs as shown in Fig. 5c; **(f)** T cell proliferation using CFSE as shown in Fig. 6a; **(g)** Cytokine production after PMA/ionomycin stimulation as shown in Fig. 6b; **(h)** Splenic T follicular helper (T_{FH}) cells as shown in Fig. 7a; **(i)** Splenic germinal center (GC) B cells as shown in Fig. 7b; **(j)** $CD4^+$ T cell frequncies and cytokine production after PMA/ionomycin stimulation as summarized in Fig. 8e and 8f, respectively; **(k)** Cytokine production after PMA/ionomycin stimulation as quantified in Fig. 9d.

Supplementary Tables

Supplementary Table 1. Antibodies for flow cytometry

Antigen	Clone	Manufacturer	Conjugation
B220	RA3-6B2	eBioscience	FITC
CD4	GK1.5	eBioscience	PacificBlue, PE, FITC, APC
CD8a	53-6.7	eBioscience	PE, PacificBlue, APC
CD19	MB19-1	eBioscience	PacificBlue
CD25	PC61.5	eBioscience	APC
CD38	90	eBioscience	APC
CD44	IM7	eBioscience	FITC
CD62L	MEL-14	eBioscience	APC
CXCR5	SPRCL5	eBioscience	PerCP-Cy5.5
Foxp3	FJK-16s	eBioscience	PE
GL.7	GL7	eBioscience	FITC
IFNγ	XMG1.2	eBioscience	APC, PE
IL-17A	eBio17B7	eBioscience	FITC, APC
IL-4	11B11	eBioscience	PE
PD-1 (CD279)	RMP1-30	eBioscience	APC
ORAI1	2C1.1	Amgen	none
ORAI1	29A2	Custom (Immunogenes)	none

Supplementary Table 2. Primers for qRT-PCR

Gene name	Forward primer	Reverse primer
mouse *Stim1*	ATTCGGCAAAACTCTGCTTC	GGCCAGAGTCTCAGCCATAG
mouse *Stim2*	TCGAAGTGGACGAGAGTGATG	TTTCCACTGTTTCCACAAATCC
mouse *Orai1*	AGACTGCCTGATCGGATGGC	TTGTCCCCGAGCCATTTCCT
mouse *Orai2*	GCAGCTACCTGGAACTCGTC	GTTGTGGATGTTGCTCACCG
mouse *Orai3*	CAGTCAGCACTCTCTGCGG	TGGCCACCATGGCGAAG
mouse *Hprt*	AGCCTAAGATGAGCGCAAGT	TTACTAGGCAGATGGCCACA
human *ORAI1*	GATGAGCCTCAACGAGCACT	ATTGCCACCATGGCGAAGC
human *ORAI2*	TGGCGGAAGCTCTACCTGAG	CGGGTACTGGTACTGCGTC
human *HPRT1*	ACCCTTTCCAAATCCTCAGC	GTTATGGCGACCCGCAG

Supplementary Table 3. Primers for site-directed mutagenesis

Mutation	Forward primer	Reverse primer
ORAI1 E106A	gtggcaatggtggcggtgcagctggac	gtccagctgcaccgccaccattgccac
ORAI1 L273D	gacagttccaggaggacaacgagctggcgg	ccgccagctcgttgtcctcctggaactgtc
ORAI2 E80Q	ccagctgcacctgcaccatggccac	gtggccatggtgcaggtgcagctgg
